# Polycaprolactone/polyacrylic acid/graphene oxide composite nanofibers as a highly efficient sorbent to remove lead toxic metal from drinking water and apple juice

**DOI:** 10.1038/s41598-024-54969-y

**Published:** 2024-02-22

**Authors:** Mohammadreza Rostami, Gholamreza Jahed-khaniki, Ebrahim Molaee-aghaee, Nabi Shariatifar, Mahmood Alizadeh Sani, Mahmood Azami, Sima Rezvantalab, Soghra Ramezani, Marjan Ghorbani

**Affiliations:** 1 Division of Food Safety and Hygiene, Department of Environmental Health Engineering, School of Public Health, Tehran University of Medical Sciences, Tehran, Iran; 2 Food Science and Nutrition Group (FSAN), Universal Scientific Education and Research Network (USERN), Tehran, Iran; 3 Department of Tissue Engineering, School of Advanced Technologies in Medicine, Tehran University of Medical Sciences, Tehran, Iran; 4 Department of Chemical Engineering, Urmia University of Technology, 57166-419, Urmia, Iran; 5 Faculty of Textile Engineering, Urmia University of Technology, 5716693188, Urmia, Iran; 6 Nutrition Research Center, Tabriz University of Medical Sciences, Tabriz, Iran

**Keywords:** Electrospinning, Polycaprolactone, Graphene oxide, Heavy metal, Lead removal, Environmental sciences, Materials science

## Abstract

Due to the characteristics of electrospun nanofibers (NFs), they are considered a suitable substrate for the adsorption and removal of heavy metals. Electrospun nanofibers are prepared based on optimized polycaprolactone (PCL, 12 wt%) and polyacrylic acid (PAA, 1 wt%) polymers loaded with graphene oxide nanoparticles (GO NPs, 1 wt%). The morphological, molecular interactions, crystallinity, thermal, hydrophobicity, and biocompatibility properties of NFs are characterized by spectroscopy (scanning electron microscopy, Fourier transform infrared spectroscopy, X-ray diffraction, Thermogravimetric analysis), contact angle, and MTT tests. Finally, the adsorption efficacy of NFs to remove lead (Pb^2+^) from water and apple juice samples was determined using inductively coupled plasma optical emission spectroscopy (ICP-OES). The average diameter for PCL, PCL/PAA, and PCL/PAA/GO NFs was 137, 500, and 216 nm, respectively. Additionally, the contact angle for PCL, PCL/PAA, and PCL/PAA/GO NFs was obtained at 74.32º, 91.98º, and 94.59º, respectively. The cytotoxicity test has shown non-toxicity for fabricated NFs against the HUVEC endothelial cell line by more than 80% survival during 72 h. Under optimum conditions including pH (= 6), temperature (25 °C), Pb concentration (25 to 50 mg/L), and time (15 to 30 min), the adsorption efficiency was generally between 80 and 97%. The adsorption isotherm model of PCL/PAA/GO NFs in the adsorption of lead metal follows the Langmuir model, and the reaction kinetics follow the pseudo-second-order. PCL/PA/GO NFs have shown adsorption of over 80% in four consecutive cycles. The adsorption efficacy of NFs to remove Pb in apple juice has reached 76%. It is appropriate and useful to use these nanofibers as a high-efficiency adsorbent in water and food systems based on an analysis of their adsorption properties and how well they work.

## Introduction

Heavy metals (HMs) are chemical elements with atomic weights between 63.5 and 200.6 Dalton and a specific gravity of over 5.0. They are a significant subcategory of food contaminants, originating from naturally occurring events, industrial operations, and environmental pollutants^1^. Toxic HMs and metalloids, like arsenic (As), lead (Pb^2+^), cadmium (Cd), and mercury (Hg), are unnecessary for biological processes and have expanded globally, posing significant risks to the ecosystem and human health^2,3^. The high prevalence of toxic HMs in the food chain creates significant health risks due to their non-biodegradability, stability, bioaccumulation capacity, and high toxicity levels. These metals accumulate rapidly in the human body through biomagnification in food chains, and even in small amounts, they are known to cause cancer^1^. Lead pollution negatively impacts cognitive development, leading to neurological and cardiovascular disorders in humans, especially in children^4,5^. Moreover, HMs like Pb^2+^ and Cd are carcinogenic and can cause bone fractures, cardiovascular issues, renal dysfunction, hypertension, liver, lung, brain, and immune system disorders^3,6^.

To reduce and eliminate HMs, researchers have developed various methods to address the inevitable presence of heavy metals in food and water, including coagulation, ion exchange, adsorption, electrostatic interaction, chemical precipitation, advanced oxidation, membrane separation, and membrane filtration^7,8^. Adsorption is preferred for eliminating HMs due to its various adsorbents, notable efficacy, simplicity, reversibility, and cost-effectiveness^9–11^. Adsorption is a popular method for removing HMs from aqueous samples due to its efficiency, ease of implementation, reversibility, and cost-effectiveness. Common adsorbents include activated carbon, natural zeolites, nanofibers (NFs), graphene, and nano-metal particles^12–16^. However, nanofibrous materials, with their large surface area and porosity, offer enhanced adsorption capacity and faster rates. They can be easily separated or recycled post-adsorption, making them effective adsorbents for HMs^16–18^.

Several techniques, like electrospinning, self-assembly, phase separation, solvent thermal synthesis, and the template method, are currently used for fabricating NFs ^19–21^. Electrospinning is a cost-effective technique in this field, offering efficient, straightforward operation and equipment. The resulting nanomaterials, known as electrospun NFs, have a large specific surface area, excellent porosity, favorable permeability, modifiable pore structure, and easy functionalization^22–25^.

Polymers are suitable for developing NFs as adsorbents, which have proper mechanical and physical properties and also have functional groups suitable for the adsorption of heavy metals. On the other hand, easy and large-scale production are also significant factors in choosing the type of polymers. Polycaprolactone (PCL) is a versatile polymer with excellent mechanical strength, flexibility, and a high surface area to volume ratio, with a slow degradation rate and easy customization for specific applications^26,27^. Also, Polyacrylic acid (PAA) is a significant polyelectrolyte substance that possesses extensive usage as a complexing agent for metal ions, stabilizers in nanoparticle production, and as a component in polyelectrolyte multilayer building^28^. PAA is a biodegradable polymer that offers multiple benefits for eco-friendly usage.

PAA has carboxyl groups (–COOH) in its chemical composition, which can create complexes with heavy metal ions via ion-exchange processes^29^. The carboxyl groups can form complexes with heavy metal ions such as Pb, Cd, or Hg; therefore, efficiently extracting them from aqueous solutions^30^. Under acidic conditions, PAA exists in its protonated state and has a greater attraction towards heavy metal ions.

On the other hand, graphene oxide (GO) has a planar structure composed of a solitary layer of carbon atoms organized in a hexagonal lattice. This configuration offers a substantial expanse, enabling a notable capacity for adsorbing heavy metal ions^31^. The surface of GO is adorned with oxygen-containing functional groups, including hydroxyl and carboxyl groups^32,33^. These functional groups can engage with heavy metal ions via electrostatic interactions, π-π stacking, and coordination bonds, resulting in a high adsorption affinity.

GO is characterized by its chemical stability, environmental compatibility, and little impact on ecosystems^32,33^. Additionally, it may be easily regenerated and reused to remove heavy metals. Furthermore, it demonstrates selectivity in adsorbing heavy metals. It has been discovered that it has a greater attraction towards heavy metal ions, such as Pb, Cd, and Hg, in comparison to other metal ions^32–34^.

**Figure 1:**
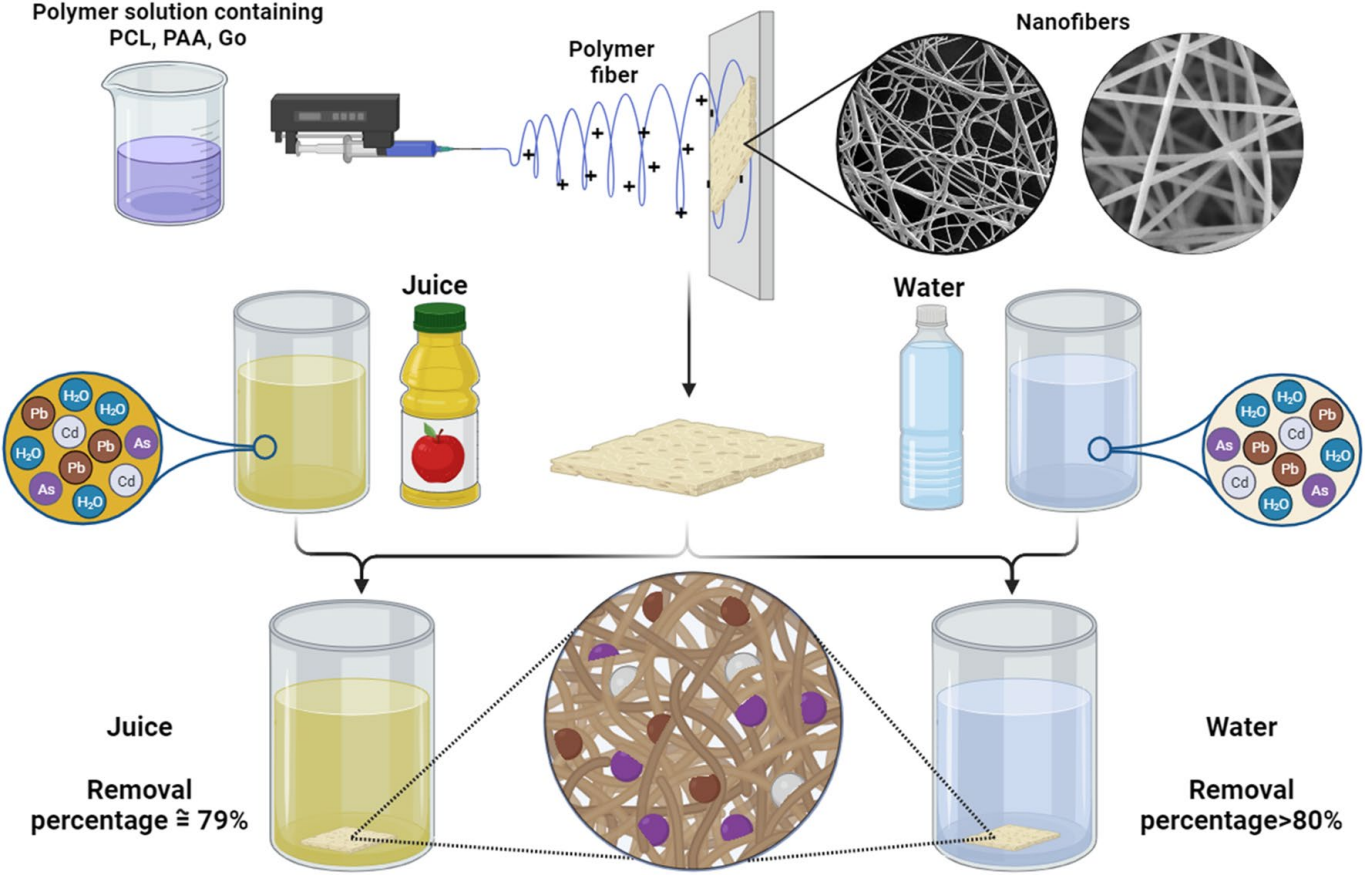
Schematic of the preparation of PCL/PAA/GO nanofibers using the electrospinning method and their application in absorbing lead metal from water and apple juice.

In this research, a PCL/PAA/GO composite nanofiber is produced, in which PCL has been considered due to having suitable mechanical and physical properties, as well as PAA and GO, to increase the maximum adsorption efficiency. We conducted the absorption of lead heavy metal in the water environment and utilized this adsorbent nanocomposite for the first time to adsorb lead metal in apple juice and food systems (Fig. 1).

## Materials and methods

### Materials

Polycaprolactone (80000 Mn/g mol^−1^), polyacrylic acid, and lead (II) nitrate were purchased from Sigma Aldrich (St. Louis, USA). Acetic acid, ethanol, and chloroform were acquired from Sigma Aldrich (St. Louis, USA). The single-layer graphene oxide was obtained from Amin Bic Company (Tehran, Iran). The MTT test kit, including 3-(4,5-dimethylthiazol-2-yl)-2,5-diphenyltetrazolium bromide, was purchased from Gibco (GIBCO Invitrogen GmbH, Germany). A selection of commercial apple juices from various brands (Tehran, Iran) were acquired for adsorption analysis. All solvents were analytical grade, and deionized water was used for all experiments.

### PCL/PAA/GO composite NFs preparation

A solution of PCL in a concentration of 12% w/v (intrinsic viscosity 0.9Z/cm^3^ g^−1^, surface tension 35.5 mN/m) was prepared in acetic acid (90%) and was used for the preparation of NFs. Fibers were created by adding PAA (1 wt%) and PCL (12 wt%). GO (1 wt%) was added to a solution containing PCL (12 wt%), and this mixture was employed to create fibers. Lastly, a blend consisting of PCL (12 wt%), PAA (1 wt%), and GO (1 wt%) has been prepared and developed into fiber structures. Subsequently, the fibers that have been generated are assessed and described.

To carry out the electrospinning process, the prepared polymer solution is placed in the syringes of the feeding pump of the device (Electroris ES1000 FNM Co., Tehran, Iran), and the flow rate of the device is set at 1 ml/h. The final voltage set for fiber preparation was 25 kV. Also, the distance between the needle and the collector was 10 cm, and an aluminum foil on the drum collector (500 rpm) was used to collect the fibers. All the stages of fiber preparation were under ambient temperature and pressure conditions, and the relative humidity of the environment was 50%.

### Characterizations and measurements

#### Morphology and structure

The 2 × 2 cm pieces of prepared NFs were subjected to scanning electron microscopy (SEM) (Philips XL 30 FEG, Amsterdam, Holland) at 26 kV to indicate their morphology and diameter. All prepared NFs are covered with a thin layer of gold before SEM. Image J software was used to measure the diameter of the prepared NFs, and the average diameter of the fibers and their frequency were analyzed using Origin Pro 2022 (V9.9.0).

#### Molecular interactions

Fourier transform infrared spectroscopy (FTIR) (Tensor27, Bruker Co., Ettlingen, Germany) has been used to investigate the molecular interaction of NFs. The spectra were recorded within the wavenumber range of 500 to 4000 cm^−1^ with a spectral resolution of 4 cm^−1^. Pellets for the samples were prepared using KBr powder.

#### Crystallinity

The X-ray diffraction (XRD) patterns of the nanofiber mats were obtained using an X-ray diffractometer (Bruker AXS, Karlsruhe, Germany) at room temperature. The measurements were conducted within the 2θ angle range of 5° to 70°.

#### Thermal stability

A thermogravimetric analysis (TGA, Germany, Linseis, STA PT1600) has been used to investigate the weight reduction and thermal stability of NFs. This test has been conducted under atmospheric N_2_ conditions and in the temperature range of 25 ºC to 700 ºC.

#### Surface wettability

The Data Physics OCA 20 contact angle system was used to compute contact angles for NFs, with each measurement completed with 10 μL of distilled water, and the results averaged based on three different scenarios.

### Thickness and mechanical property

The NFs' thickness was measured using a thickness gauge (C640, Labthink) at five randomly selected points on each sample. The thickness of each sample was determined by calculating the average of the collected values. The tensile strength, elongation at break, and Young's modulus of the generated NFs were determined using a Tensile Analyzer (Instron 5566, USA) by ASTM D-882. The specimens were sectioned into fragments of 5 mm × 30 mm. The experiments were conducted at ambient temperature and a velocity of 0.04 mm/s^35^.

### Porosity, surface areas, and in vitro biodegradation studies

The porosity of the generated NFs was determined using the liquid displacement technique (Eq. 1)^36^.

1 $$\mathrm{Porosity }\left(\mathrm{\%}\right)=\frac{{{\text{V}}}_{1}-{{\text{V}}}_{3}}{{{\text{V}}}_{2}-{{\text{V}}}_{3}} ,$$

V1 and V2 denote the original volume of ethanol (96%) and the volume after immersing nanofiber samples, respectively. Additionally, V3 reflects the amount of ethanol after extracting the nanofibers, which occurs after 5 min.

To evaluate the degradation behavior in a laboratory environment, electrospun samples with a surface area of 1 cm^2^ were submerged in a solution of PBS (pH 7.4) for 7 days, with the temperature of the solutions maintained at 37 ± 1 °C. The specimens were extracted from the solutions at different time intervals (1, 2, 3, 4, 5, and 6 days) and then rinsed with distilled water, dried by exposure to air, and measured to determine the percentage of weight reduction. The weight loss of the NFs mat was determined using Eq. (2):

2 $$\mathrm{WL }\left(\mathrm{\%}\right)=\frac{{{\text{W}}}_{{\text{i}}}-{{\text{W}}}_{{\text{f}}}}{{{\text{w}}}_{{\text{i}}}}\times 100$$

Let *W*_*i*_ represent the starting weight and *W*_*f*_ represent the weight of the NFs mat after it has dried for various days. The Brunauer–Emmett–Teller (BET) technique was used to compute the specific surface areas. The pore volume and pore size were determined using the Brrett-Joyner-Halenda (BJH) technique.

### Biocompatibility study

The biocompatibility of the NFs was assessed against human umbilical vein endothelial cells (HUVEC) using the MTT cell proliferation reagent. The NFs were prepared by cutting them into round discs with a diameter of 15 mm. Subsequently, the discs were sterilized and carefully arranged on a 24-well plate. Then, a volume of 1 mL of cell suspension, including 1 × 10^5^ cells in each sample, was added and subjected to incubation for durations of 24, 48, and 72 h at a temperature of 37 °C and an atmosphere containing 5% carbon dioxide. Subsequently, the wells holding the samples were loaded with 400 μL of dimethyl sulfoxide solvent and subjected to a further four-hour incubation period. The supernatant was optically examined for the presence of insoluble purple formazan products using the ELISA microplate reader (Elx808, Biotek, USA) at a wavelength of 570 nm. The control group in this study consisted of HUVEC cells.

### Adsorption study in water

In this study, composite nanofiber (1 mg) was mixed with Pb^2+^ solution (100 mg/liter, 30 mL) to investigate adsorption efficacy from water solutions. Factors such as pH, interaction time, temperature, and Pb^2+^ concentration were optimized for effective removal. The samples were shaken at 150 rpm for varying times, and the NFs were extracted from the solution. The ICP-OES device was used to determine the remaining Pb^2+^ in the solution. The tests were conducted in duplicate and the average results were reported. The percentage of metal ion removal was determined using Eq. (3).

3 $$\mathrm{\%Removal}=\frac{{{\text{C}}}_{{\text{I}}}-{{\text{C}}}_{{\text{e}}}}{{{\text{C}}}_{{\text{e}}}}\times 100,$$

where C_i_ and C_e_ represent the initial and ultimate concentrations of the metal ion in solution, measured in milligrams per liter (mg/L), respectively.

### Adsorbent selectivity

The study assessed the selectivity of PCL/PAA/GO composite NFs in a specific concentration of HMs like Pb^2+^, Cd, and Hg. The experiment was conducted under optimized conditions including pH = 6, temperature = 25 °C, and an adsorbent dosage = 1 mg. The ICP-OES equipment was used to evaluate the solution and determine the degree of removal for each of the remaining dissolved HMs.

### Regeneration and recycling

The PCL/PAA/GO NFs composite underwent adsorption, desorption through rapid filtration, and multiple washes with distilled water to determine its reusability. After that, 50 ml of 0.1-M HCL solution was added to the adsorbed Pb^2+^/adsorbents, and the mixture was stirred for 2 h. Finally, the solution was filtered to separate the nanofibers. Subsequently, the nanofibrous material was subjected to a drying process in an oven at a temperature of 60 °C for 2 h. Before the second adsorption, the adsorbent underwent treatment with a 0.1 M NaOH solution for 2 h. The regenerated NFs were introduced into the Pb^2+^ solution for the adsorption process.

### Molecular dynamics simulations

A number of model simulations were created in the current work to simulate the interactions between Pb^2+^ ions, modified GO, and polymers. All simulations were based on weight percentages of components in the total compositions to produce simulations that were comparable to wet-lab environments. Since surface modification of GO with amine results in the formation of GOHNH_2_, we prepare and parameterized GOHNH_2_ using Polypargen webserver^37^. Moreover, the GOHNH_2_ component in the acidic environment would be protonated and converted to GOHNH^3+^, this component has been modeled in the acidic solutions.

On the other hand, the structure of both polymers cannot be changed in the acidic solutions. Therefore, the structure of PAA and PCL polymer chains was prepared using Charm-GUI polymer builder^38^. Finally, the topology of all components was obtained using the Charmm-GUI Ligand Reader and Modeler^39^ with Charmm-36 m force field^40^. Then, all-atom molecular models with multiple molecules solvated in water were prepared using Charmm-GUI Multicomponent Assembler^41^. In this section, the addition of Pb^2+^ ions was carried out with neutralization of simulations with PbCl_2_ salt.

Subsequently, GROMACS 2020 was used to further stabilize the simulations by using periodic boundary conditions (PBCs) in the EM (with a minimum force of 10 kJ/mole/nm), NVT (for 500 ps with time steps of 1 fs), and NPT (for 500 ps with time steps of 1 fs). The study used the particle mesh Ewald (PME) method^42^ to handle long-range electrostatic interactions, the isotropic Parrinello-Rahman algorithm^43^ to control the pressure at 1 bar, and the Berendsen thermostat to regulate the temperature at 300 K. The hydrogen bonds were restricted using the LINCS algorithm^44^. Throughout the process, the van der Waals and Coulomb interactions have been taken into account, with a cutoff radius of 1.4 nm. Ultimately, molecular dynamics simulations were conducted for a duration of 100 ns, using a time step of 2 femtoseconds. Additional analysis of the simulations was conducted with the GROMACS program. In addition, the models were visualized using VMD software^45^.

### Adsorption of lead metal by NFs in apple juice media

The clear and pulp-free apple juice samples were used to quantify the initial concentration of Pb^2+^ metal. Along with the apple juice sample as a control (unspiked sample), a Pb^2+^ standard solution (100 ppm, 100 µL) was added to the apple juice samples. Accordingly, NFs (1 mg) were also added to all samples. The juice samples were subjected to acid digestion, and the quantity of Pb^2+^ metal was measured using ICP-OES equipment.

### Statistical analysis

For every experiment, the study examined each condition in triplicate, and the results were presented as means and standard deviations (SD). Using Origin 2022 software, one-way ANOVA and Tukey’s test were used to examine variations in the results. The threshold for statistical significance was established at P < 0.05.

### Ethics approval and consent to participate

The writers of this paper did not conduct any investigations involving human volunteers or animals.

## Results

### Morphology and structure

**Figure 2:**
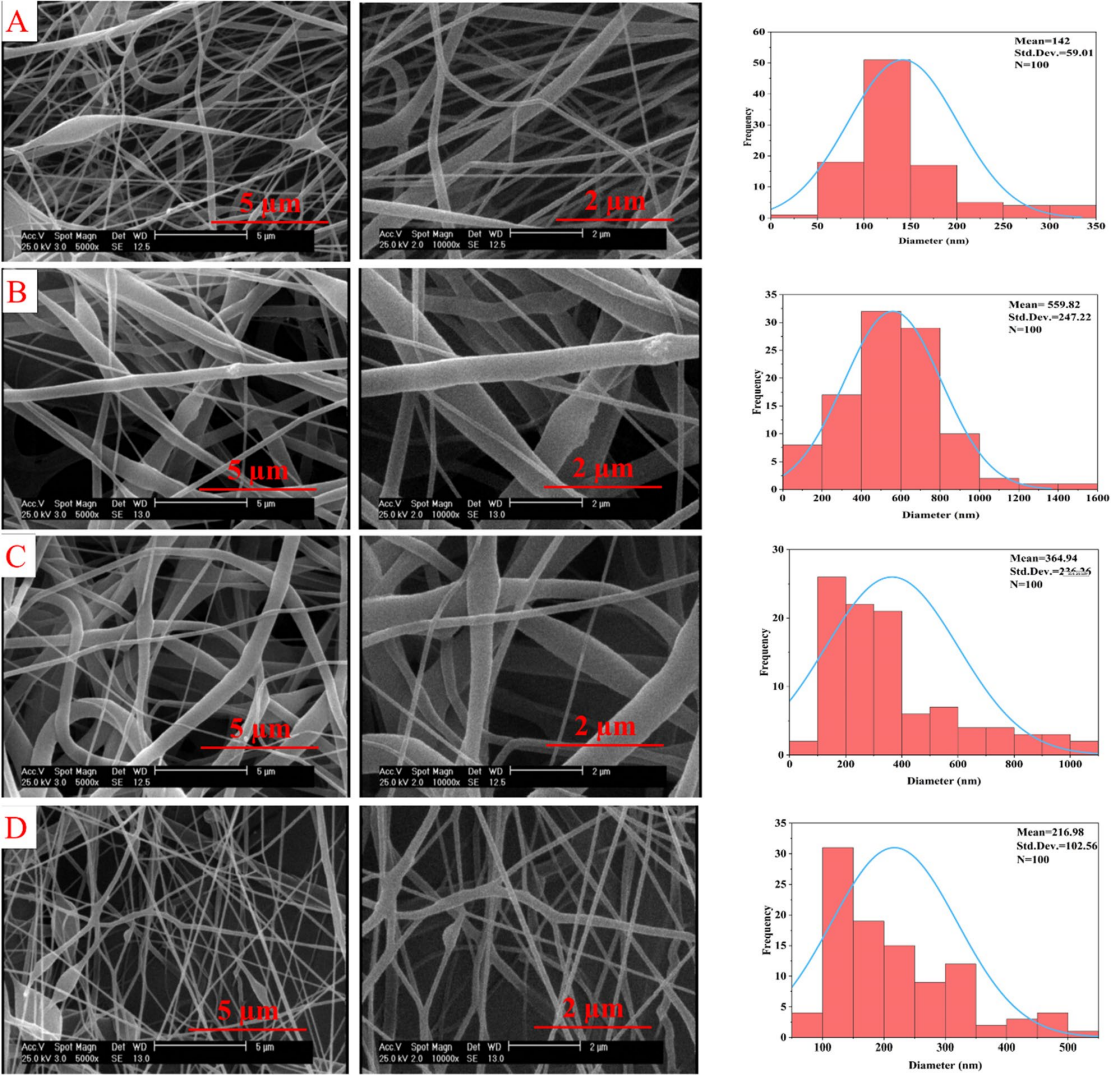
SEM images at magnifications of 2 and 5 µm and analysis of the distribution and average diameter of NFs (**A**) PCL, (**B**) PCL/PAA, (**C**) PCL/GO, and (**D**) PCL/PAA/GO.

Our investigation found that PCL solution has the optimum conditions for fiber production at a concentration of 12% w/v. At this weight concentration, fibers can be easily separated from the surface of the collector. In this concentration, the ejection of beads from the needle tip was less observed. To maintain the optimum conditions and also to maintain the ability to produce fibers, PAA polymer was added to the composition at a concentration of 1% wt, and the results showed that with the addition of PAA to the PCL composition at a concentration of 1% wt, there was no change in the ability to produce fibers. With the addition of GO nanoparticles at a concentration of 1%, there was no change in the ability to produce fibers. Figure 2 shows the SEM images of the shape of PCL-based NFs in various stages.

SEM pictures and the analysis of PCL NFs using Image J software reveal that this polymer has an average diameter of 137 nm when presented at a concentration of 12% w/v (Fig. 2A). As seen in the images, the highest concentration of chain entanglements and overlap concentration for this polymer is seen at this particular concentration. Indeed, this concentration yielded the greatest rate of fiber synthesis in our study^22,24^.

By including PAA in the PCL composition at a concentration of 1% w/v, the uniformity of fiber production remains consistent. However, there is a noticeable upward trend in the average diameter of the NFs, as shown in Fig. 2B. The SEM image analysis revealed that the NFs had an average diameter of 500 ± 247 nm. The enlargement of the fibers’ diameter may be attributed to the rise in the viscosity of the intended solution for fiber formation^46^. Figure 2C shows an observed increase in the number of beads in the NFs when PCL is combined with GO. The conducted analysis revealed that the average diameter of the NFs in this chemical was 364 ± 236 nm. The fibers produced in this composition have a wide range of diameter sizes, but a greater distribution of the average diameter has occurred in the range between 150 and 500 nm.

Various researches have shown that the inclusion of GO in nanofiber mat results in an augmentation of solution conductivity, thus leading to an enlargement of fiber diameters^46–48^. The final mixture consisting of PCL/PAA/GO exhibits an increased presence of beads on the fibers. The average diameter of the fibers in this mixture was 216 ± 100 nm, as shown in Fig. 2D. The mean fiber diameter exceeds that of PCL alone, although falls below that of other compounds when coupled with PCL.

Based on the analysis of 100 fibers using Image J software, it has been discovered that the majority of fibers have a maximum diameter ranging from 100 to 300 nm. Findings positively correlate with previous studies. Raj et al*.*^49^ showed that the combination of PCL and gelatin produces a band with a size ranging from 260 to 500 nm. Also, in another study, where uniform and beadles fibers have been produced, it was shown that the production of fibers based on PCL ranged from 200 to 600 nm in average fiber diameter^50^. In other studies, it has been observed that with the addition of secondary polymers to the PCL composition, an increase in the average diameter of the fibers occurs^51^. Oner et al*.* produced PCL/PAA to be used as an electrochemical sensor. The optimum combination of a PCL/PAA concentration ratio of 9:1 has been reported. The average diameter of nanofibers for this ratio was reported to be 259.4 nm^52^.

### Molecular interactions

**Figure 3:**
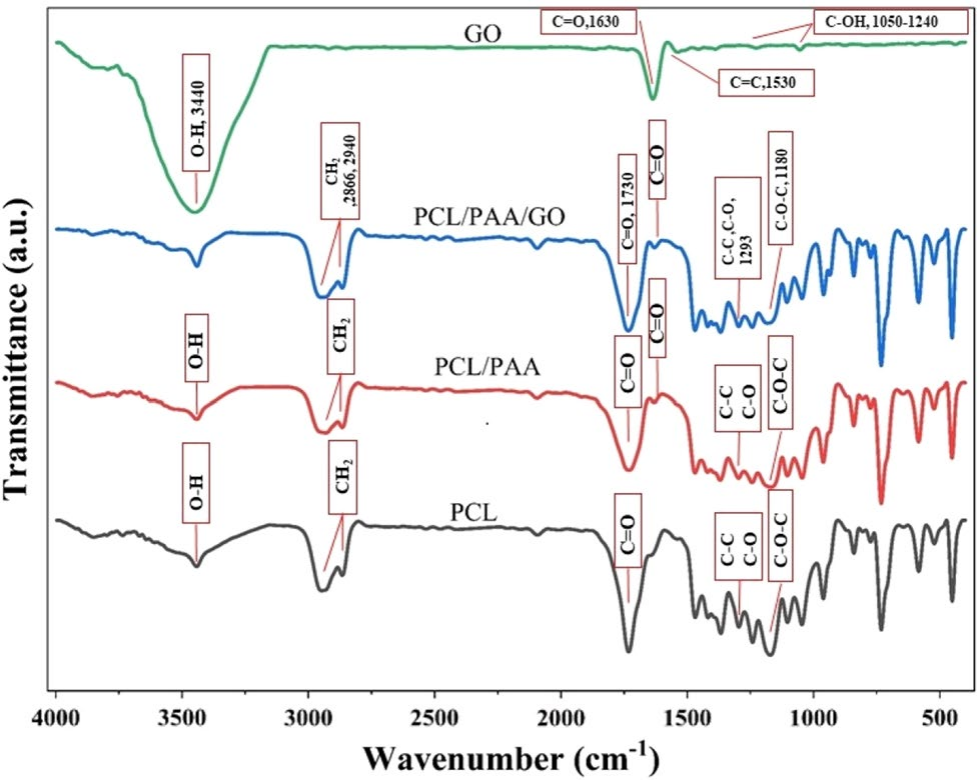
FTIR spectra of GO, PCL, PCL/PAA, and PCL/PAA/GO composite NFs.

FTIR is a non-destructive analytical technique used to identify and analyze the functional groups presented in a wide range of materials^53^. FTIR analysis can provide valuable insights into the chemical interactions and bonding presented within the PCL, PCL/PAA, GO, PCL/PAA/GO nanofibers structure (Fig. 3). The functional groups presented in PCL may be identified by analyzing its FTIR spectrum, which exhibits the following peaks.

The presence of an asymmetric -CH_2_ bond is shown by the adsorption peak at 2943 cm^−1^. The compound exhibits a symmetric –CH_2_ stretching vibration at 2866 cm^−1^, a carbonyl stretching vibration at ~ 1722 cm^−1^, C–O and C–C stretching vibrations at 1293 cm^-1^, and an asymmetric C–O–C stretching vibration at ~ 1180 and 1239 cm^−1^. The presence of water in the nanofibers' composition causes the existence of O–H stretching vibrations, which is attributed to the peak area of 3440 cm^−1^^52,54^. These findings are parallel with previous studies.

In a study, Ramirez et al.^55^ used PCL in combination with whey protein to remove chromium from water. The results obtained in this study are consistent with the spectrum obtained in FTIR for the composition of PCL NFs^55^. The FTIR spectrum also includes the addition of PAA to the PCL mixture. Additionally, the spectrum exhibits peaks associated with PCL polymer. The spectrum displays peaks corresponding to the carbonyl group in the ~ 1630 cm^−1^, which is not observed in the PCL spectra. This peak serves as a confirmation of the existence of PAA in the composition of PCL/PAA NFs. The band seen at 3282 cm^−1^ corresponds to the stretching of O–H bonds in PCL and exhibits a little reduction with adding PAA to the composition. Also, the peak in the ~ 1250 cm^−1^ region is related to the C–O stretch, which is presented in both spectra, but the intensity of the peaks is reduced due to the presence of PAA in the composition^56^.

The identification of oxygen functionalities in GO was determined based on the existence of peaks corresponding to the C=O stretching peak at 1630 cm^−1^, C=C stretching at 1530 cm^−1^, and C–OH (hydroxyl) stretching at 1050–1240 cm^−1^^57^. The range between 3100 and 3800 cm^−1^ corresponds to the presence of OH groups in carboxyl and H_2_O structures^58^.

As shown in the results, in the final composition of PCL/PAA/GO, all peaks and spectra in other samples are also observed. The area peak at 1630 cm^−1^ has verified the existence of PAA. The PCL NFs alone do not reveal this peak. This peak is also seen for GO, and it indicates its presence in the final composition. The results of FTIR show that the carbonyl groups were in the region of 1732 cm^−1^ for PCL, which shifted to 1727 cm^−1^ with the addition of PAA. Also, with the addition of GO to the final composition, this amount has increased to 1736 cm^−1^.

The PCL composition shows the peak related to OH groups in region 3443 cm^−1^. It can be seen that with the addition of PAA, it has shifted to 3439 cm^−1^, and with the addition of GO, this value has decreased to 3440 cm^−1^. The FTIR results for the nanofibers of the final composition showed the shift of most of the peaks in their respective regions^54,59^. The peaks obtained in this study are compatible with similar studies^50^. Palacios et al*.* produced PCL/nanocellulose nanofibers for use in heavy metal filtration. They pointed out that within the PCL spectrum, there are prominent bands, such as the carbonyl (C=O) group, which exhibit a stretching vibration at around 1723 cm^−1^. The peaks seen at 2954 and 2859 cm^−1^ are indicative of the asymmetric and symmetric stretching of CH_2_. The bands seen at 1473, 1243, and 1176 cm^−1^ correspond to the bending of C–C bonds, the stretching of asymmetric C–O–C bonds, and the stretching of symmetric C–O–C bonds, respectively. The peak seen at 1291 cm^−1^ represents the stretching of C–O and C–C bonds in the crystalline phase, whereas the peak at 1157 cm^−1^ corresponds to the stretching of C–O and C–C bonds in the amorphous phase^60^.

Oner et al*.* prepared PCL/PAA nanofibers containing pyronose oxidase to be used as sensors. The peaks of each material alone are the same as the peaks of the compounds in our research. In this research, no specific interaction between PCL and recombinant PAA was observed^52^.

Huang et al. produced PCL/PAA nanofibers for use as wound dressings. The peaks obtained in the FTIR spectrum for this research were similar to the previous studies, with the difference that a new peak related to hydroxyl groups was created in the region of 300 to 3600 cm^−1^^61^.

### Crystallinity

Based on Bragg’s law, which proves that X-rays diffracted by atoms generate a diffraction pattern, XRD is a potent method used to evaluate material crystal structure and phase composition^62^. By analyzing this pattern, it can be determined the crystal structure, lattice parameters, and phase composition of the material under investigation^62^. In the case of PCL/PAA/GO NFs, XRD can provide valuable insights into the arrangement of atoms and the presence of specific phases within the nanofiber structure. Understanding the crystal structure of these NFs is crucial for tailoring their properties and optimizing their performance.

**Figure 4:**
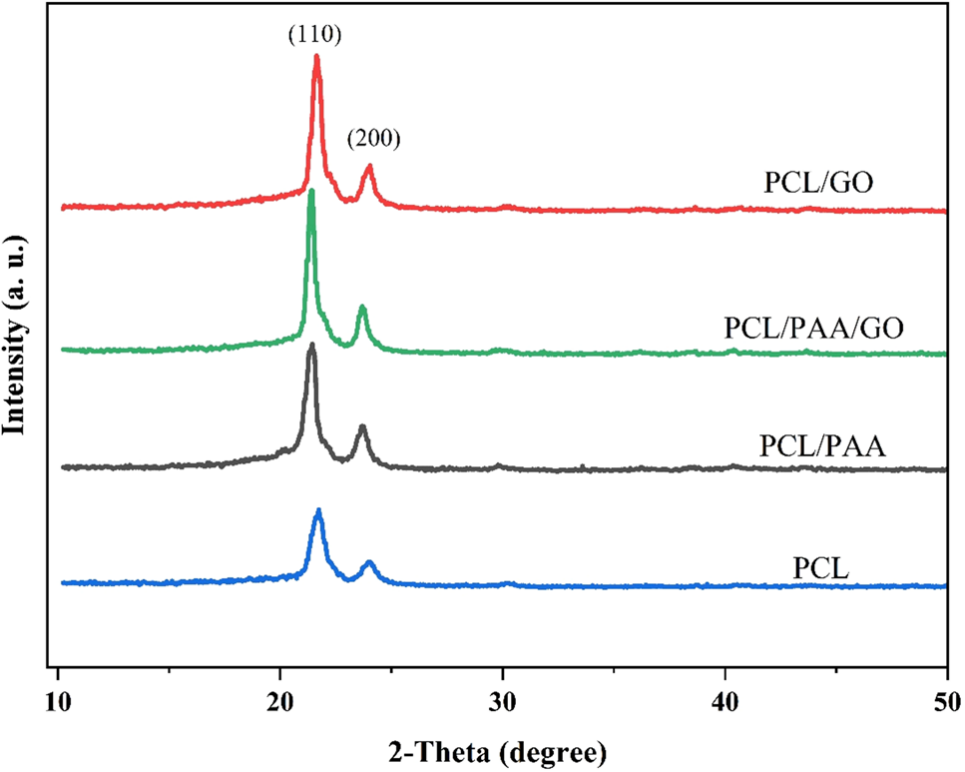
XRD pattern of PCL, PCL/PAA, PCL/GO, and PCL/PAA/GO composite NFs.

PCL is a biodegradable polyester that exhibits a semi-crystalline structure^63^. The XRD analysis of PCL-based NFs typically shows diffraction peaks at 2θ = 21.69° and 23.94° corresponding to the (110) and (200) planes of the orthorhombic crystalline phase (Fig. 4)^64^. The position and intensity of these peaks provide valuable information about the crystal structure and crystallinity of the nanofiber. However, adding PAA/GO to the PCL matrix, the intensity of the peaks fairly increased, suggesting the proper compatibility and the influence of PAA/GO on the increase of the crystallinity and phase composition of NFs^65^. The addition of GO to the composition of PCL causes a very small increase in the amount of nanofiber crystallinity. This has also been reported in other studies^65–67^. According to the XRD information, the mixed fiber appears to have a semi-crystalline structure. In the study by Roberto et al.^68^, the addition of GO to the PCL composition did not result in any discernible peak. The reason for this is the low concentration of GO in the composition^68^.

### Surface wettability, porosity, mechanical properties, and in vitro biodegradation studies

**Figure 5:**
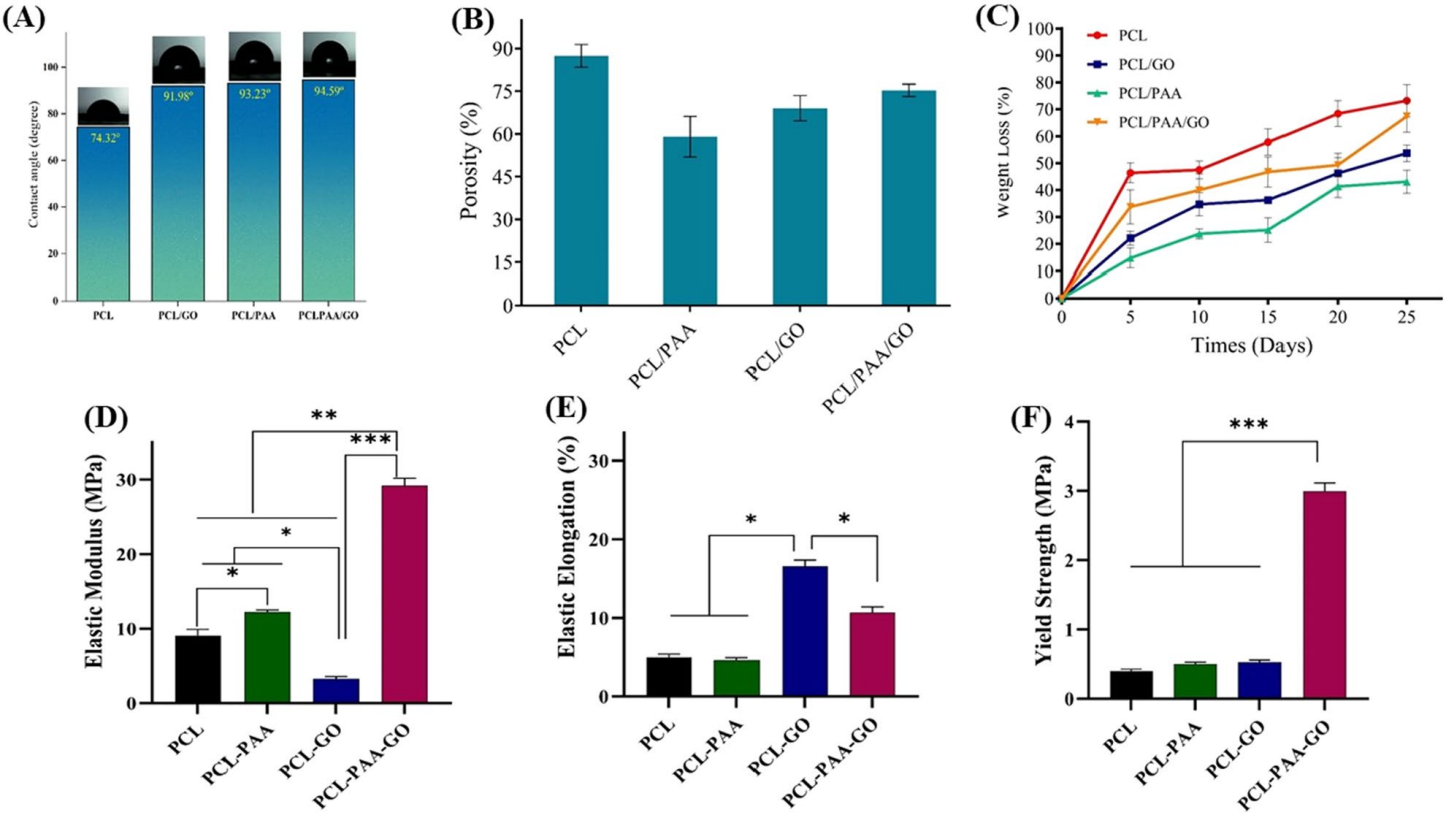
Evaluation of the characteristics of nanofibers using tests of (**A**) water contact angle, (**B**) porosity, (**C**) degradation rate, and (**D**–**F**) mechanical properties of PCL, PCL/PAA, PCL/GO, and PCL/PAA/GO nanofibers, respectively.

The contact angle test was conducted to quantify the hydrophobic properties of the developed NFs (Fig. 5A). Typically, when the contact angle is large and the drop deposited on the fiber surface retains its spherical form, the compound exhibits more hydrophobicity. The findings indicate that PCL fibers possess inherent hydrophobic characteristics. Specifically, the contact angle for ethene NFs was measured at 74.32º, demonstrating a distinct hydrophobic behavior. The hydrophobicity of PCL is attributed to the presence of CH_2_ groups throughout the primary polymer chain^69^.

The hydrophobicity of the final nanofiber increases as the proportion of hydrophilic polymers in the mixture decreases. The experimental findings indicate that the addition of PAA to the PCL composition enhances the hydrophobic nature of the NFs. Furthermore, NFs have seen an augmentation in their hydrophobic characteristic, and the contact angle of nanomaterials has surpassed the threshold of 90º. The hydrophobicity or hydrophilicity of NFs is influenced by their porosity and average diameter^70^. This research demonstrates that augmenting the mean diameter of the fibers and the proportion of PAA in the PCL composite enhances its hydrophobic properties.

Despite the water-soluble and hydrophilic qualities of PAA polymer, its incorporation has led to a notable increase in the diameter of the fibers, increasing the hydrophobicity of the composite fiber. Indeed, when the average diameter of the NFs decreases, the surface-to-volume ratio of the NFs increases, resulting in enhanced hydrophilic characteristics of the composition. Moreover, augmenting the mean diameter of NFs leads to a reduction in the ratio of surface-to-volume, hence enhancing the hydrophobic nature of the NFs^70^.

The incorporation of GO into PCL has resulted in an augmentation of the hydrophobic nature of the resulting nanofiber. As shown in the morphological discourse, the incorporation of GO into PCL resulted in an augmentation of the fiber diameter. Nevertheless, this quantity is smaller than the one in PAA. In several research, the inclusion of GO has been shown to enhance their hydrophilic property^47^. However, this particular study reveals that the level of hydrophobicity in fibers is mostly influenced by their width. When it comes to using NFs for adsorbing heavy metals in water and fruit juice, it is advantageous because the NFs are hydrophobic and cannot dissolve in water. Conversely, it will be more convenient to isolate it from liquid systems after adsorption. The investigation conducted by Yavari et al*.*^71^ validates the findings of our study regarding the analysis of the contact angle. Also, it was found that the contact angle for PCL polymer was 78.9° in a study by Sachin et al*.*^72^. This polymer, along with other hydrophobic polymers, raised the desired value. Oner et al*.* reported a water contact angle of 72.9 for the PCL/PAA nanofiber composite. which was due to the increase in the amount of PAA in the composition^52^.

Porosity is one of the outlining characteristics of NFs in the adsorption of heavy metals. Obviously, NFs with high porosity show higher adsorption. As shown in Fig. 5B, the porosity of each NF of PCL, PCL/PAA, PCL/GO, and PCL/PAA/GO is 87.5, 59.1, 69.1, and 75.36, respectively. The results of the porosity measurement are consistent with the results obtained from the SEM. As it has been shown, PCL NFs have the highest amount of porosity, while this composition has the lowest average diameter of NFs. Additionally, the average diameter of NFs explains why PCL/PAA NFs have the lowest porosity.

Adding GO to the PCL composition, the amount of porosity has been reduced. It can be seen that the porosity in the PCL/PAA/GO composition is slightly different from that of PCL NFs. This is also related to the average diameter of the fibers. All the factors that cause the production of fibers in a uniform form and also cause the change in the fibers’ diameter are far more effective in determining the amount of porosity.

The degradation of NFs has been investigated for 25 days. The addition of different compounds and their degradation are shown in Fig. 5C. Fibers with high porosity, low nanosized, and a high surface-to-volume ratio show a faster degradation rate^73^. As can be seen in the degradation test results, the composition of PCL is degraded to a greater extent, and an upward trend in the degradation of these fibers is observed. Hence, 73.1% of PCL nanofibers were destroyed within 25 days. The fibers that have the least amount of porosity are less damaged during this period. However, the amount of destruction for PCL/PAA, PCL/GO, and PCL/PAA/GO nanofibers was 43, 53.66, and 67.36%, respectively. Although PCL nanofibers are expected to degrade later than other compounds, it has been observed that this does not happen. Likely, the presence of higher porosity and a higher surface-to-volume ratio in the PCL composition is the reason for the earlier degradation of this composition.

The nitrogen adsorption technique was used to investigate the specific surface area and pore structure of the composite NFs. Table 1 shows the values of the specific surface area, the total porosity, and the size of the porosity for each of the nanofiber compositions. According to the results, it can be seen that by adding PAA to PCL, there is a significant decrease in the amount of specific surface and total porosity, as well as the size of pores. As seen in the SEM results, PCL has the lowest average fiber diameter and the highest specific surface area. However, in the final mix of PCL, PAA, and GO, the specific surface area has gone down by the same amount that the average fiber diameter has grown.

**Table 1:** The composite nanofibers possess characteristics such as BET surface area, total volume of pores, pore diameter, and thickness.

Sample	BET (m^2^ g^−1^)	Pore volume (cm^3^ g^−1^)	Average pore diameter (nm)	Thickness (µm)
PCL	81.925	0.117	5.7141	75
PCL/PAA	10.069	0.01824	7.2462	30
PCL/GO	33.446	0.046401	5.5494	105
PCL/PAA/GO	42.393	0.060563	5.7143	55

Moreover, the thickness of nanofibers is shown in Table 1. The mechanical properties of the samples are shown in the Fig. 5D–F. The composition of the samples influences their mechanical properties. The addition of PAA to PCL increases its Young’s modulus while adding GO to PCL decreases it. Notably, simultaneous addition of GO and PAA to the PCL composition significantly enhances Young's modulus. This increase in Young's modulus of PCL/PAA/GO composite results in a much higher yield stress compared to other samples. Additionally, the tensile stress-induced elongation of the material is also influenced by adding additives to PCL. While the addition of PAA has little effect on elastic elongation, incorporating GO increases elongation to 16.8%. Simultaneous addition of PAA and GO results in a composite with an elastic elongation of 10.4%, representing a significant difference compared to PCL. Overall, the addition of PAA and GO to the composite has enhanced its mechanical properties. This improvement is more pronounced when both PAA and GO are added to the sample simultaneously.

### Thermal stability

**Figure 6:**
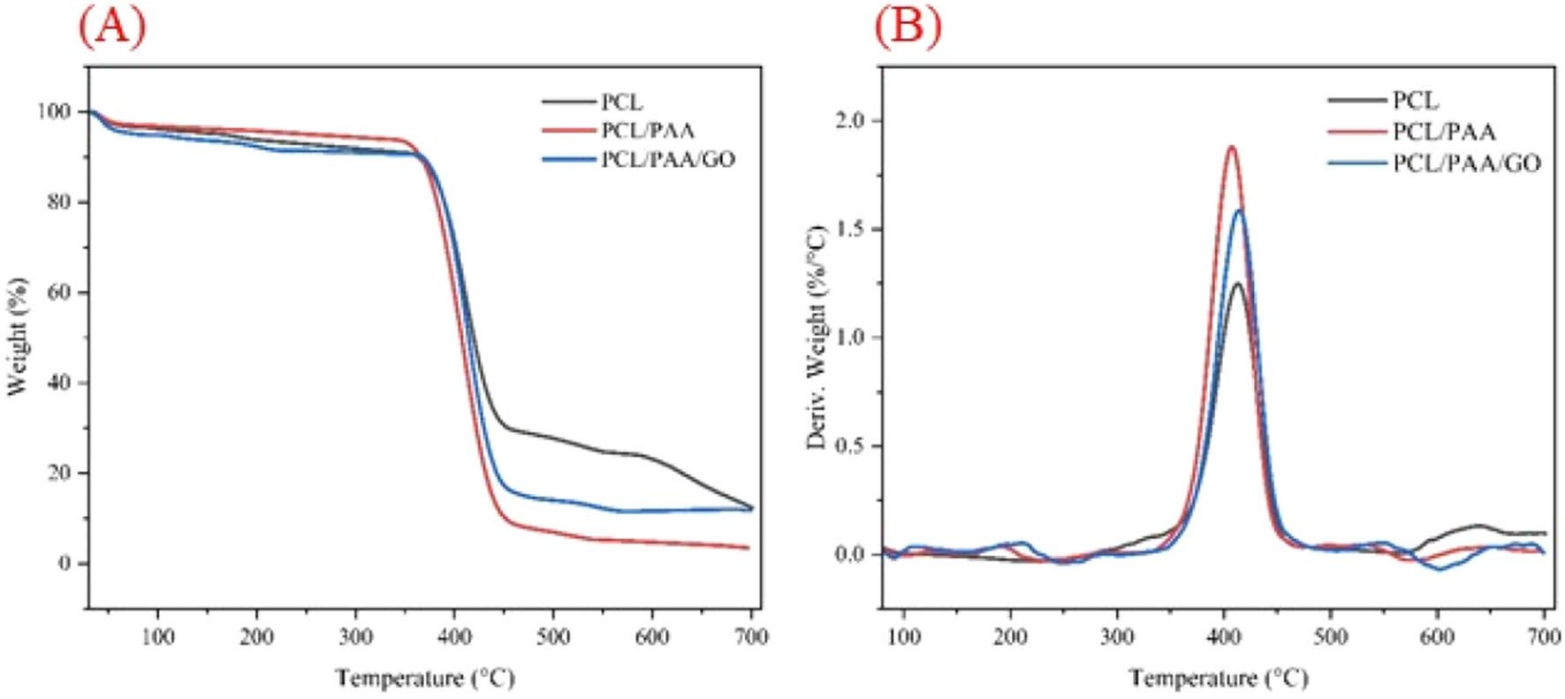
Thermal analysis of the prepared NFs. (**A**) Thermogram of weight loss percentage in the temperature range of 25 to 700; (**B**) Weight derivative according to temperature.

The thermal stability of produced NFs has been assessed using TGA. The study was conducted within the temperature range of 25 to 600 ºC, as shown in Fig. 6. The TGA thermogram demonstrates that three sets of produced fibers have a comparable and closely aligned thermal stability behavior. A marginal decrease in weight is detected across the three groups when exposed to temperatures ranging from 25 to about 390 ºC. This weight reduction, which accounts for 7 to 10 % of the total weight, may be attributed to the evaporation of moisture from the NFs. The thermogram of PCL indicates no alteration until reaching a temperature of 370 ºC, after which a notable decrease in weight occurs at temperatures over 400 ºC.

The presence of PCL NFs in the composition results in a significant decrease in weight within the temperature range of 370 to 450 ºC. Approximately 70% of the NFs have experienced decomposition when PCL NFs are heated from 450 to 700 ºC. Despite being exposed to a temperature of 700 ºC, the ultimate weight of the NFs stays at 10%. The graph demonstrates that the inclusion of PAA in the PCL composition diminishes the stability of the NFs that are produced.

Prior research on the thermal examination of PCL has shown that 70% of PCL is decomposed at a temperature of 450 ºC^55^. Out of all the nanofiber composites, the PAA/PCL composite exhibits the most rapid degradation rate. According to the graph, the compound's weight decreases to just 9% when exposed to a temperature of around 450 ºC. Nevertheless, other nanofiber compositions have shown greater durability. The PCL/PAA/GO NFs exhibit an initial susceptibility to heat, similar to other NFs, resulting in a destructive response. The onset of significant degradation occurs at a temperature of 370 ºC, with the most substantial weight reduction seen between 370 and 450 ºC. Nevertheless, it is important to acknowledge that within this specific temperature range, the carbonyl groups found in GO experience deterioration.

Ultimately, it is evident that fibers including GO exhibit greater stability compared to PCL/PAA fibers within the temperature range of 450 to 700 ºC. The uppermost heat limit for the deterioration of NFs is shown in Fig. 6B. The most significant nanofiber degradation takes place within the temperature range of 400 to 420 in all three nanofiber groups. Within this specific temperature range, it is well-established that the PCL/PAA fibers had the most significant level of degradation. The PCL fibers exhibit lower levels of degradation within this temperature range. Previous investigations have shown a consistent outcome, where electrospun NFs with the highest proportion of PCL exhibited the least amount of weight loss and the greatest level of thermal stability^74–76^. Previous studies showed the same results. In a study conducted by Ran et al.^50^, PCL nanofibers had the highest stability against heat. With the addition of other materials to PCL, its thermal stability decreases. Also, in other studies, a temperature of about 400 °C results in the highest amount of nanofiber destruction^50^.

### Biocompatibility

**Figure 7:**
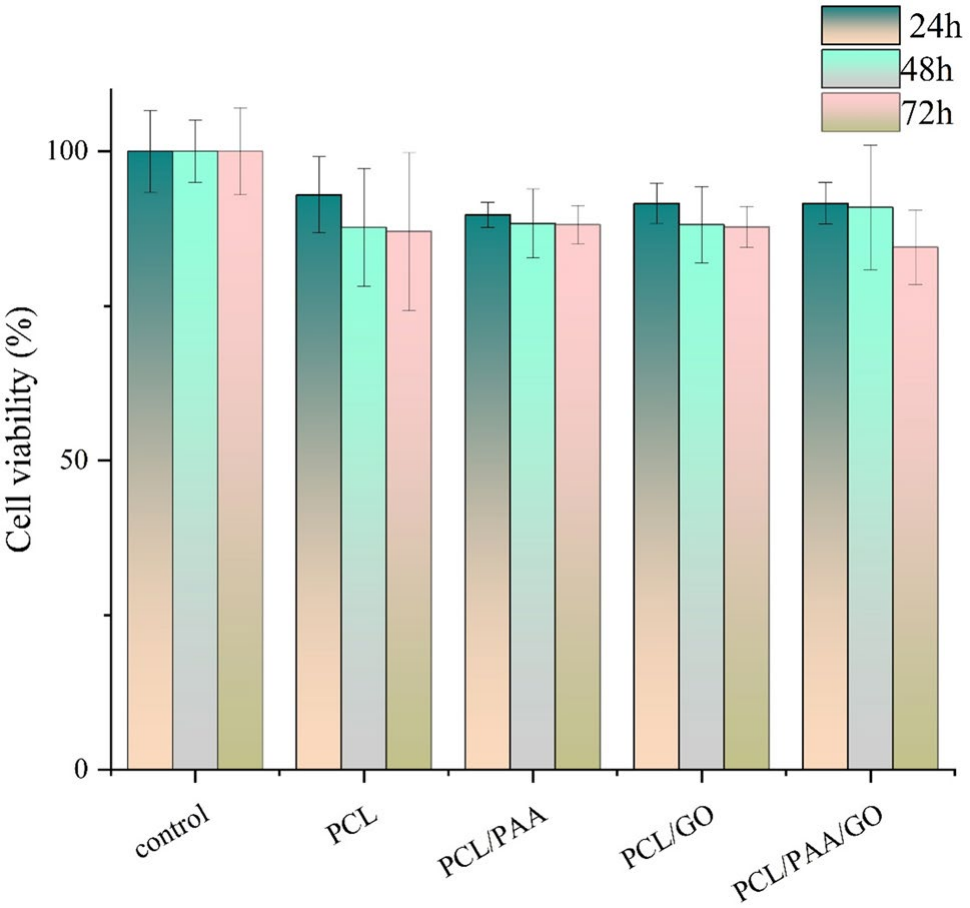
Cytotoxicity for PCL, PCL/PAA, and PCL/PAA/GO NFs in HUVEC cells for 24, 48 and 72 h.

The cytotoxicity level of NFs is crucial for their use in food and food-related systems. The cytotoxicity of PCL, PCL/PAA, and PCL/PAA/GO NFs in HUVEC cells was assessed at 24, 48, and 72 h, as seen in Fig. 7. As seen in the illustration, none of the nanofiber groups exhibited cytotoxicity. The PCL/PAA/GO group had the lowest survival rate of the groups listed, with a rate of 84.4% after 72 h. Indeed, alternative fibers have shown a superior rate of survival. In other studies, PCL NFs containing GO have increased the proliferation of cells^47^. In these studies, the cause of this increase in growth has been attributed to increased hydrophilicity, antibacterial properties, and the ability to adsorb protein. Prior research has shown that PCL fibers not only lack cytotoxic effects but promote cell growth^71,77,78^. The non-toxicity of the investigated NFs confirms their applicability in food-related systems.

### Adsorption study

Electrospun NFs show significant promise as adsorbents for heavy metals and other contaminants^16^. These materials possess a large specific surface area, significant porosity, and excellent functional capacity, and may be transformed into mats or membranes to effectively eliminate contaminants from water-based solutions^79^. Furthermore, they may be easily separated or regenerated after the process of adsorption. Therefore, nanofibrous materials have been regarded as very effective adsorbents for heavy metals^16^.

NFs have garnered significant interest in the realm of environmental pollution management and remediation in recent years. The use of these materials in food systems, particularly in beverages such as juices, is a recent development and is regarded as current research. The current study analyzed the polymers and efficient substances that are well-suited for the adsorption of heavy metals. Ultimately, the PCL/PAA/GO composite has been used to effectively adsorb and eliminate the toxic heavy metal lead, as well as a diverse array of heavy metal mixtures under various circumstances.

**Figure 8:**
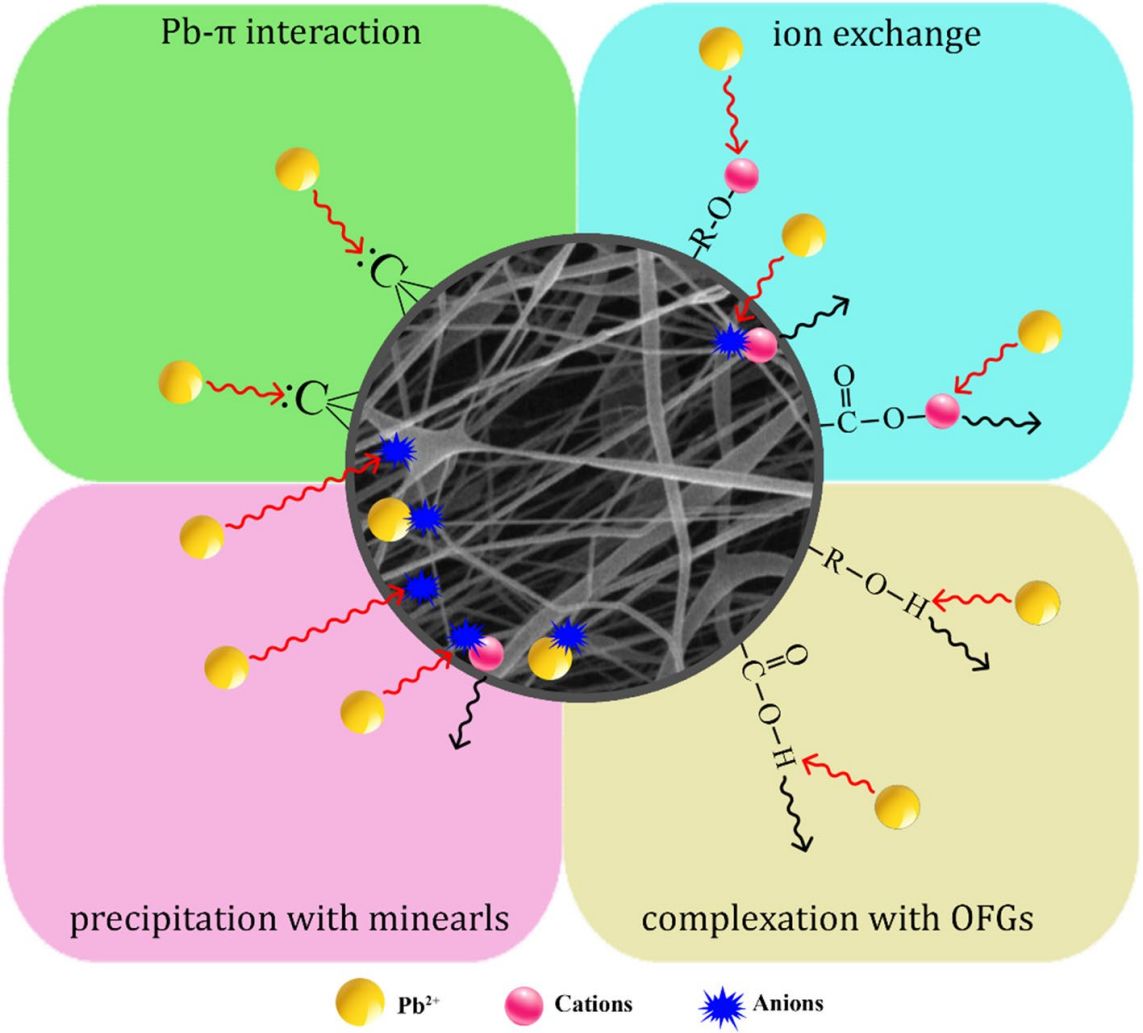
Schematic of lead metal adsorption by PCL/PAA/GO nanofibers.

Adsorption of heavy metal ions onto functional groups on the surface of NFs may be classified into two categories: chemical adsorption and physical adsorption. HMs adsorption on NFs is mostly dependent on interactions between functional groups and heavy metal ions, such as ion exchange, coordination chelation, and electrostatic contact (Fig. 8). A combination of physical and chemical mechanisms for the adsorption of Pb^2+^ metal on PCL/PAA/GO nanofibers has occurred. According to the functional groups on the surface of the nanofibers, which have also been determined in the FTIR analysis, electrostatic attraction and chemical bonds are responsible for the adsorption of Pb^2+^ metal on the surface of the nanofibers.

**Figure 9:**
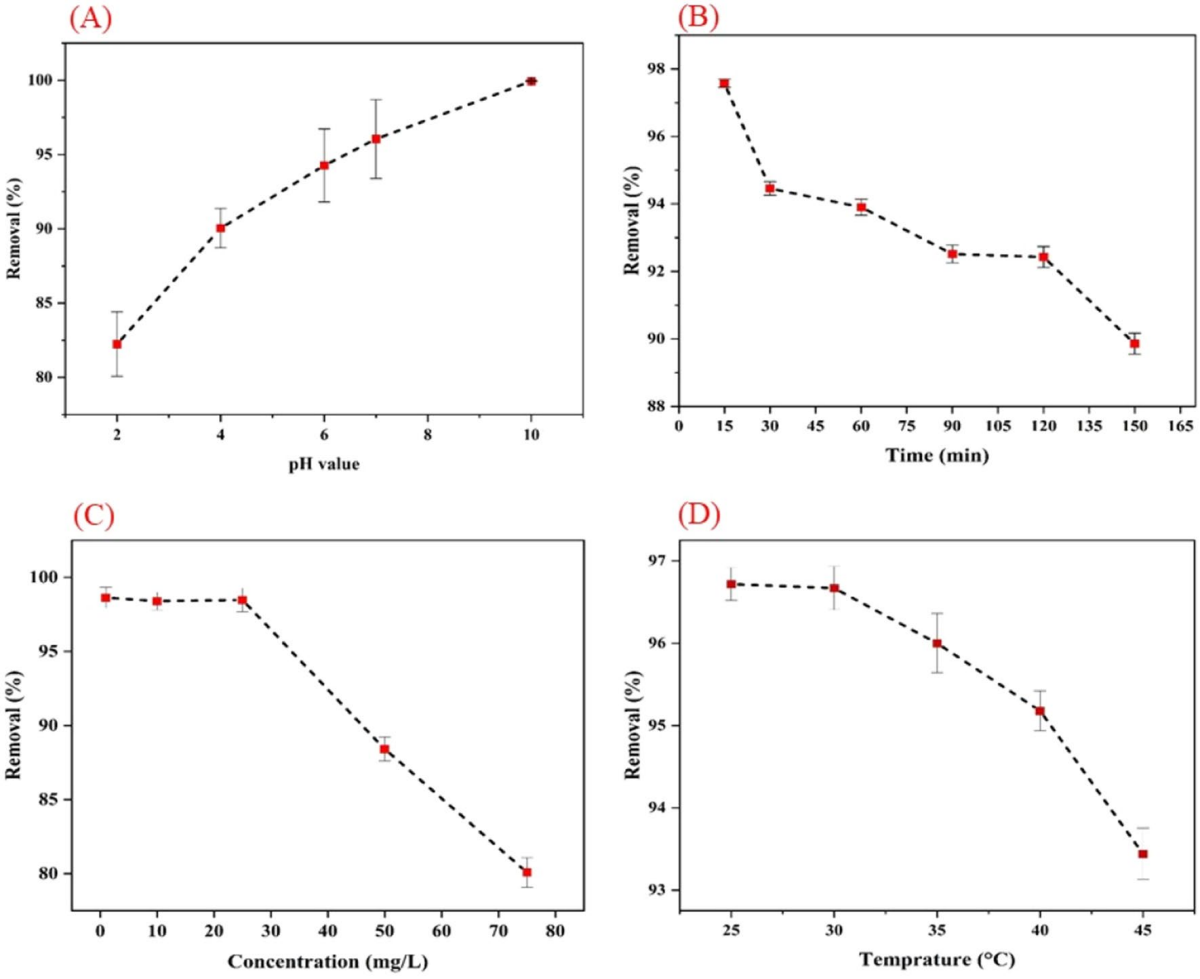
The percentage of lead heavy metal removal may be influenced by several factors, including (**A**) pH level, (**B**) duration of treatment, (**C**) concentration of the solution, and (**D**) temperature.

**Figure 10:**
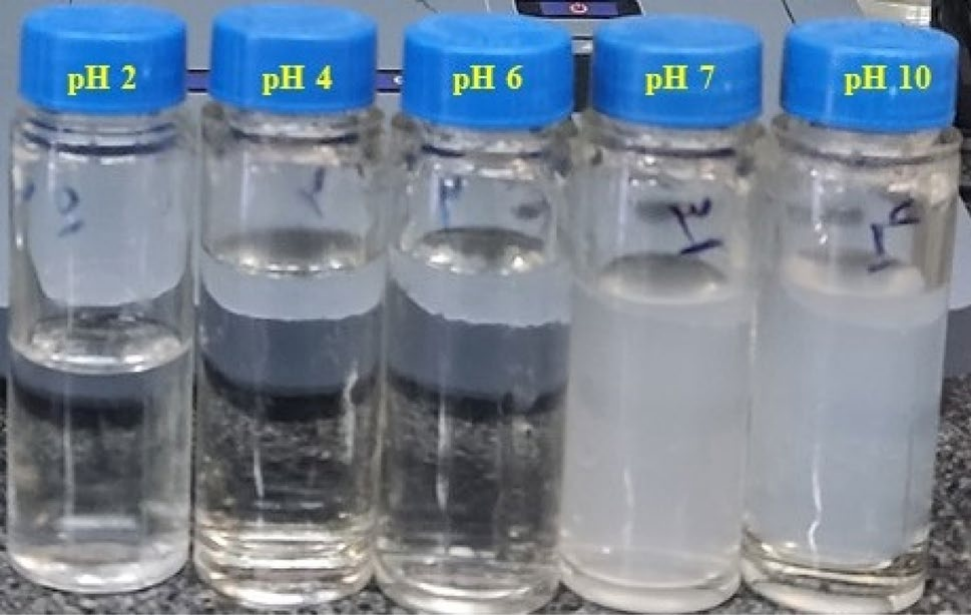
Different pH of lead nitrate for adsorption by NFs.

The conditions demonstrate the optimum level of adsorption efficiency for each element (Fig. 9). The investigation examined the impact of pH, time, temperature, and Pb^2+^ content, as seen in Fig. 9. The investigation included studying the effects of different pH levels (2, 4, 6, 7, and 10) on the removal of Pb^2+^ metal from the solution, as seen in Fig. 9A. The findings indicate that elevating the pH from a low value, such as 2, to a higher value has resulted in a rise in the adsorption of Pb^2+^ metal onto the nanofiber surface. In an acidic solution, the concentration of H^+^ and H_3_O^+^ ions is substantially larger compared to Pb^2+^ ions in the aqueous solution. Consequently, these ions may also compete with the Pb^2+^ ions for the adsorption process on the adsorbent's surface. If a substantial quantity of H^+^ and H_3_O^+^ ions attach to the adsorbent's surface, it can potentially resist the Pb^2+^ ions^80,81^. The results suggest that when the pH level is higher than neutral, namely at 7 and above, it results in the creation of solid aggregates and the aggregation of Pb^2+^ metal, as seen in Fig. 10.

This phenomenon has also been shown in other research investigations^82^. Hydroxide ions (OH^-^) are plentiful in basic solutions and readily react with lead ions (Pb^2+^) to produce Pb^2+^ hydroxide (Pb(OH)_2_), which is a compound that does not dissolve. The insolubility of lead results in its precipitation from the solution as solid particles^83^. Based on the findings and the observation that heavy metals are deposited at pH = 7 and above, pH = 6 is deemed the best level for the uptake of heavy metals. This is because the highest quantity of adsorption occurs at this pH. At a pH = 6, the functional groups undergo protonation, resulting in a reduction in the concentration of H^+^ and H_3_O^+^ ions. This drop leads to an increase in the adsorption capacity.

Time plays a crucial role in the efficient adsorption and elimination of heavy metals and contaminants. During the heavy metal removal process, it has been shown that the highest level of pollutant adsorption occurs during the first minutes of the adsorption process, as seen in Fig. 9B. This tendency has seen a consistent decline over time. This research assessed the adsorption of heavy metals during the time range of 15 to 150 min.

The impact of a 1 mg dosage of adsorbent on a solution with a concentration of 100 ppm has been examined. It was found that over 90% of the Pb^2+^ metal was adsorbed during the first 15 min. The time required for adsorption of heavy metals is directly influenced by the amount of adsorbent. As the adsorbent dose increases, its adsorption capacity also increases, resulting in a longer adsorption period for the heavy metal. Given that the adsorbent dosage is set at the minimum level and remains constant during the trial, all the adsorbent sites and capacity are fully used within the first few minutes, estimated to be 1 mg. At the start of the adsorption process, all the active sites are new and capable of adsorbing the metal ions. As a result, the removal percentage is 100% and the concentration of metal ions in the permeate is zero. Subsequently, the metal cations will cease to be adsorbed due to the eventual saturation of active sites by the ions, particularly at higher initial input concentrations^84,85^.

The concentration of Pb^2+^ is another important element in the adsorption process, as seen in Fig. 9C. The most significant reduction in Pb^2+^ metal content has occurred at quantities below 25 mg/l. Based on the minimal amount of adsorbent used, it is evident that the saturation of adsorbent sites occurs more rapidly at lower Pb^2+^ concentrations. Pb^2+^ removal from water is more efficient at lower concentrations. As the concentration of Pb^2+^ in the solution rose, the efficacy of its removal from water declined. Indeed, it is said that at low concentrations, it has the capacity to fully occupy all sites on the adsorbent, a possibility that does not arise at greater concentrations^85^.

The impact of the temperature parameter on the adsorption process has been examined, as shown in Fig. 9D. Temperatures ranging from 25 to 45 ºC have been examined and recorded for the adsorption process. As shown in this chart, the temperature of 25 ºC has the most significant impact and achieves the largest degree of Pb^2+^ metal elimination. With an increase in temperature from 25 ºC onwards, we have seen a decline in the adsorption of Pb^2+^ metal in the solution. Hence, the temperature of 25 ºC is defined as the ideal adsorption temperature in this parameter, at which the greatest adsorption value is attained.

In a study by Irandoost et al*.* on the adsorption of Pb^2+^ metal by PCL nanofibers, it was observed that pH(= 5.5), a time of 10 (min), lower concentrations (5 mg/l), and a temperature of 40 °C were the optimal points of the adsorption process, which is consistent with our results.

#### Adsorbent selectivity

**Figure 11:**
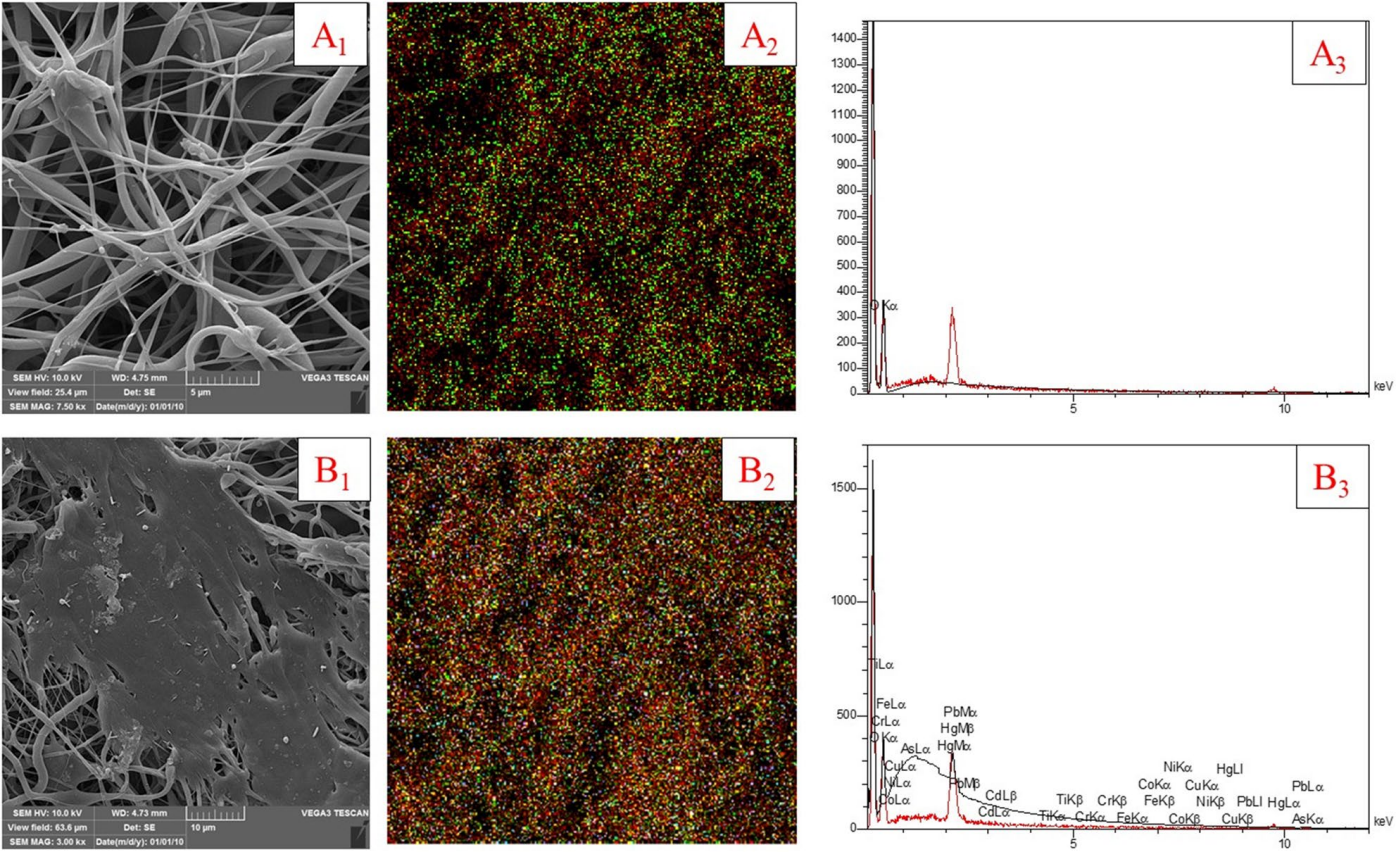
SEM of PCL/PAA/GO electrospun NFs (**A**_**1**_) before adsorption, (**A**_**2**_) mapping before adsorption, (**A**_**3**_) amount of material on fibers before adsorption and (**B**_**1**_) fiber surface after adsorption, (**B**_**2**_) mixture mapping of metals after adsorption, (**B**_**3**_) The amount of metals on the adsorbent surface.

The selectivity of heavy metals was assessed by examining PCL/PAA/GO NFs. The present investigation involves the preparation of distinct quantities of heavy metals, including lead, arsenic, cadmium, nickel, chromium, copper, iron, cobalt, and mercury. The experiment was conducted using a pH = 6, a temperature of 25 °C, and an adsorbent dosage of 1 mg. Based on SEM analysis, energy dispersive X-ray (EDX), and mapping, it has been concluded that the produced NFs have the highest affinity for mercury, followed by lead. Figure 11 illustrates the distribution of Pb^2+^ on the adsorbed membrane, obtained by EDX. This picture showcases the uniform dispersion of a significant amount of Pb^2+^ across the fibers on the surface of the nanofibrous scaffold after the adsorption process.

The consistent distribution of Pb^2+^ on the NFs indicates that the polymerization process occurred evenly along the whole surface of the fibers. Consequently, the adsorption functional groups (imide and amine) were uniformly distributed over the substrate. Observable changes in the membrane architecture may be seen after the adsorption of lead. Figure 11A1 depicts the electrospun NFs before the adsorption process, while Fig. 11A2 illustrates the spatial distribution of NFs before adsorption, and Fig. 11A3 displays the quantity of materials present before adsorption.

**Figure 12:**
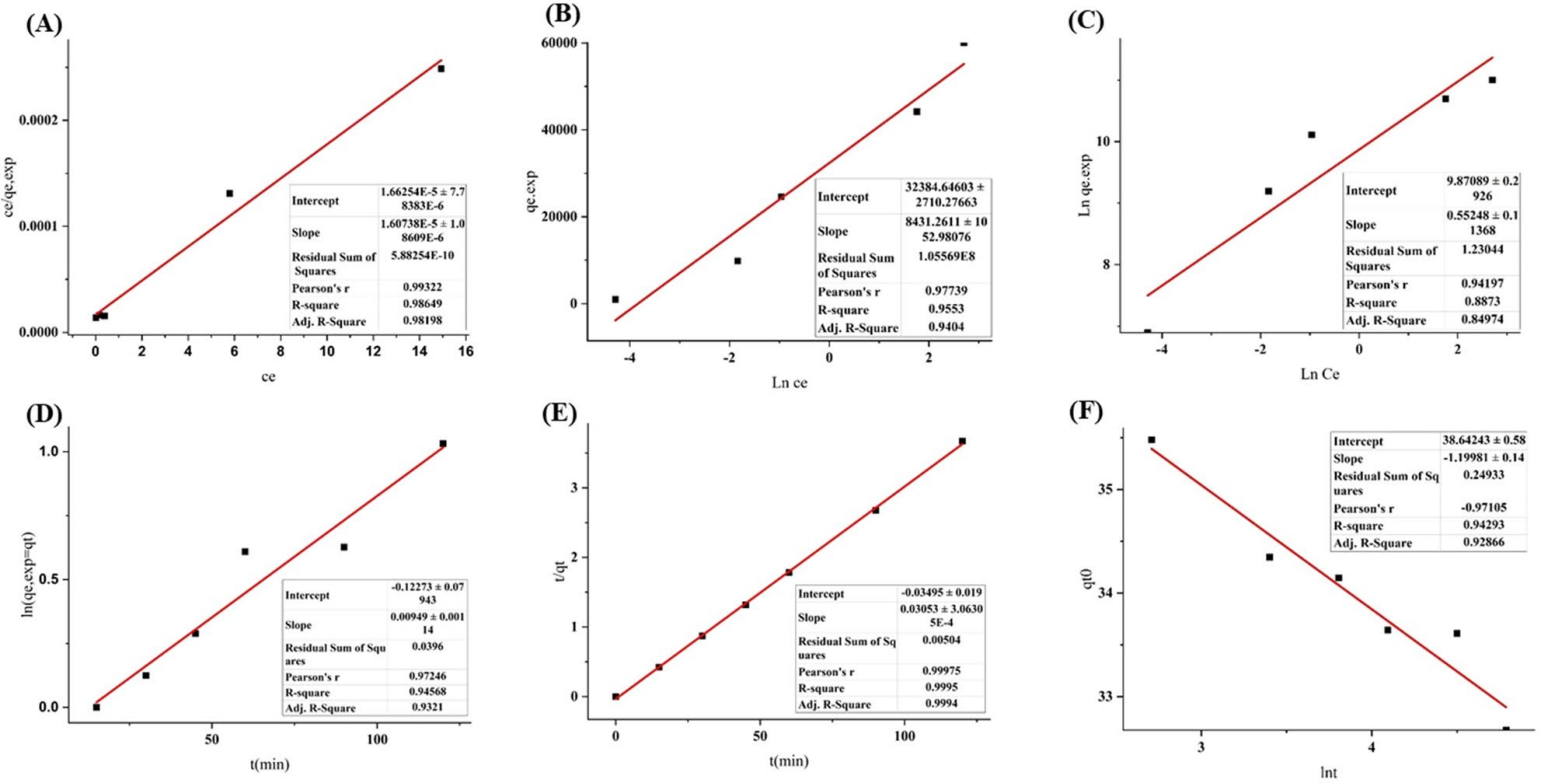
The linear curve for isotherm models (**A**-**C**) and linear curve for kinetic models (**D**–**F**) in the process of lead ion adsorption by PCL/PAA/GO nanofibers.

Moreover, Fig. 11B1 illustrates the alteration of the filamentary structure of the fibers, resulting in the formation of a sleek surface. PCL/PAA/GO NFs have been used for the sequestration and adsorption of a combination of toxic heavy metals including lead, cadmium, mercury, chromium, nickel, and copper. The mapping done on the surface of nanofibers after the adsorption process shows the dispersion of metals on the surface of nanofibers (Fig. 11B2). This dispersion is characterized by different colors on the fiber surface, which has a higher color composition than the nanofibers before adsorption. The quantity of each heavy metal is shown in Fig. 12Avia the utilization of the region under the curve. It is well-established that the greatest level of adsorption of heavy metal pollutants, namely mercury and lead, occurs when they are present in a combination of heavy metals.

#### Adsorption isotherms

Mostly the Langmuir, Freundlich, and Temkin isotherm models are used to describe the adsorption equilibrium of heavy metal ions on PCL/PAA/GO NF. The relationship between the initial concentration, temperature, and pH of the heavy metal ion solution and the amount of heavy metal ion adsorbed may be described using the adsorption isotherm^86^. The Langmuir, Freundlich, and Temkin isotherm equations were linearized using Eqs. (3), (4) and (5) respectively.

4 $${q}_{e}=\frac{{q}_{max}{K}_{a}{C}_{e}}{1+{K}_{a}{C}_{e}}$$

5 $${q}_{e}={K}_{f}{C}_{e}^{1/n}$$

6 $${q}_{e}=\frac{RT}{{b}_{T}}{\text{ln}}\left({K}_{T}{C}_{e}\right)$$

The variables in question are q_e_, which is the amount of Pb^2+^ in equilibrium (mg g^−1^), q_max_, which is the amount of Pb^2+^ ions in equilibrium (mg g^−1^), K_a_, which is the Langmuir constant (L mg^−1^), and C_e_, which is the Langmuir adsorption capacity (mg L^−1^). K_f_ represents the Freundlich constant, which is measured in units of (mg g^−1^) (L mg^−1^)^1/n^. The variable n represents the adsorption intensity. K_T_ represents the Temkin constant, which is measured in L mg^−1^. R refers to the universal gas constant, which has a value of 8.314 J mol^−1^.K^−1^. T represents the absolute temperature, measured in K. Lastly, b_T_ denotes the heat of adsorption, measured in J mol^−1^^87–89^.

The interaction between Pb^2+^ ions and PCL/PAA/GO NF is analyzed using isotherm models such as Langmuir, Freundlich, and Temkin (Fig. 12A–C). Figure 12A demonstrates that the experimental equilibrium results are best described by the Langmuir isotherm model (R^2^ = 0.98649). This indicates that there is a strong interaction between the Pb^2+^ ions and the adsorbent, likely due to the formation of a monolayer of Pb^2+^ ions on the uniform surface of the NF adsorbent. The Pearson coefficient, as shown in Fig. 12A, indicates a robust linear correlation (0.99322) between the adsorbed Pb^2+^ ion and PCL/PAA/GO NF according to the Langmuir model. Following the Langmuir absorption model, Temkin's model demonstrates more compatibility with the adsorption behavior Fig. 12B. Subsequently, the Freundlich model also exhibits consistency Fig. 12C. In Irandoost et al*.* study, Freundlich, Langmuir, and Temkin isotherm models were obtained, which is inconsistent with the results of the research.

#### Kinetic study

To find out how fast Pb^2+^ ions are taken in and how effective the adsorption process is, we compare the experimental data with pseudo-second-order, Elovich, and pseudo-first-order kinetic models. Eqs. (7), (8) and (9) were used to express linear forms of pseudo-first-order, pseudo-second-order, and Elovich, respectively.

7 $$ln\left({q}_{e}-{q}_{t}\right)=ln {q}_{e}-{K}_{1} t$$

8 $$\frac{t}{{q}_{t}}=\frac{1}{{K}_{2}{q}_{e}^{2}}+\frac{t}{qe}$$

9 $${q}_{t}=\frac{1}{\upbeta }{\text{ln}}\left(a\upbeta \right)+\frac{1}{\upbeta }{\text{ln}}t$$

Let q_e_ and q_t_ represent the quantities of Pb^2+^ ions on the PCL/PAA/GO NF at optimal conditions (mg g^−1^). The variable t represents the contact time (min), K_1_ is the constant for the PFO kinetics model (min^–1^), and K_2_ is the constant for the PSO kinetics model (g mg^−1^.min^−1^). The initial adsorption rate (mg g^−1^ min^−1^) is represented by α, while the desorption constant (g mg^−1^) is represented by β. These parameters characterize the rate of the chemisorption process^90^.

Figure 12D–F displays the linear fitting of pseudo-first-order, pseudo-second-order, and Elovich models for PCL/PAA/GO/NF adsorbents used to adsorb Pb^2+^ ions. The error regression R^2^ value of 0.9995 indicates that the adsorption process of the synthetic adsorbent is a pseudo-second-order reaction. This suggests that the adsorption process may entail many steps. The experimental equilibrium data are fitted to the PFO kinetic model (R^2^ = 0.94568) and the Elovich kinetic model (R^2^ = 0.94293) following the PSO kinetic model. The research conducted by Irandoost et al. found that laboratory data aligned with the models being used in all situations examined. Additionally, the experimental findings demonstrate an adequate match with pseudo-second-order models.

### Regeneration and recycling

To assess the durability and cost-effectiveness of the PCL/PAA/GO NFs as an adsorbent, it is crucial to examine its performance throughout four consecutive cycles of adsorption and desorption of Pb^2+^ ions onto the NFs adsorbent. This experiment aims to assess the capacity of the PCL/PAA/GO NF adsorption method to regenerate the adsorbent and its cost-effectiveness. Following the improvement of the adsorption settings, we have achieved outstanding adsorption efficiency and a remarkable capacity to regenerate.

**Figure 13:**
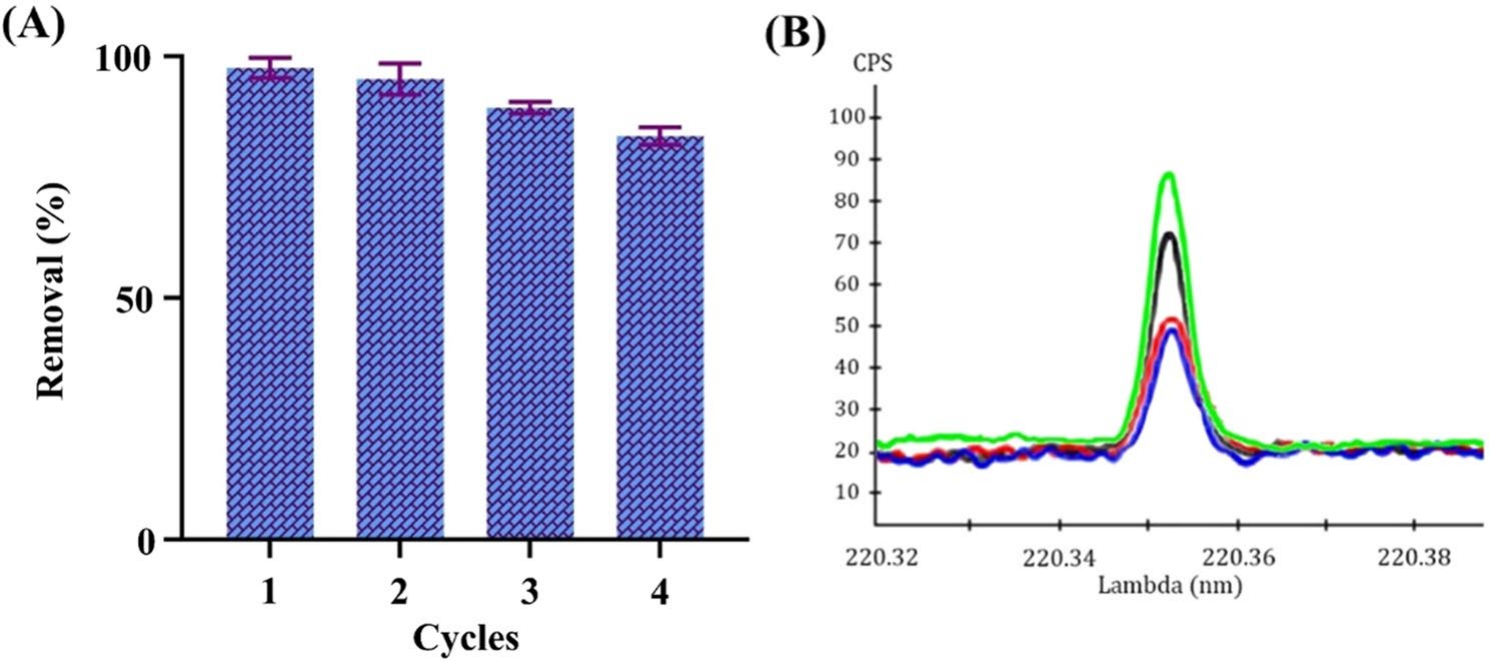
Reusability of the PCL/PAA/GO NF for Pb ion removal in (**A**) 4 cycles, (**B**) spectral peak in each cycle.

The findings indicated that the removal efficiency remained over 90% following optimization. As depicted in Fig. 13, the composite material exhibited consistent adsorption efficiency for Pb^2+^ ions even after undergoing four cycles of adsorption–desorption recycling. The efficiency remained at 97.6%, 95.3%, 89.4%, and 83.5%, respectively. As shown, with the increase in adsorption cycles, the efficiency of the adsorbent decreases and the amount remaining in the solution increases. As can be seen in Fig. 11, after the processes of adsorbing the fibrous and porous form of the nanofibers, it has become a smooth and non-porous state, which in turn causes a decrease in the active sites for the adsorption of lead metal and ultimately leads to a decrease in the adsorption efficiency in the next cycles of adsorption. This shows that the PCL/PAA/GO NFs adsorbent is very stable and can effectively get rid of recycled Pb^2+^ ions. After four stages of adsorption and desorption, the removal efficiency of heavy metals has decreased by about 15%. The slight decrease in the adsorption efficiency is due to some Pb^2+^ ions leaving the surface of the composite. The surface functional charge groups of PCL/PAA/GO NFs change when they are regenerated using an alkaline solution for desorption. This makes the Pb^2+^ ion rejection less effective. These polymers showed high potential in multi-stage adsorption. Since these polymers are not expensive, they are abundant, and the production of fibers from them is easy, the use of this system in industry can be explored.

### Molecular dynamics simulations

The use of mathematical simulations is a prevalent method for discovering logical reasons for the reported experimental outcomes. Molecular dynamics (MD) simulations are a sophisticated technique that offers enhanced understanding and in-depth analysis of experimental findings^91^. This study examines the individual and combined impact of PCL, PAA, and GO polymers on the adsorption of Pb^2+^ metal under both neutral and acidic pH conditions. We used MD modeling to examine the adsorption of Pb^2+^ metal inside these polymers and compounds. The polymer concentrations used in the simulation were identical to those utilized in the laboratory experimentation. The simulation demonstrated that the polymers in the molecule remain unchanged when exposed to acidic environments.

**Figure 14:**
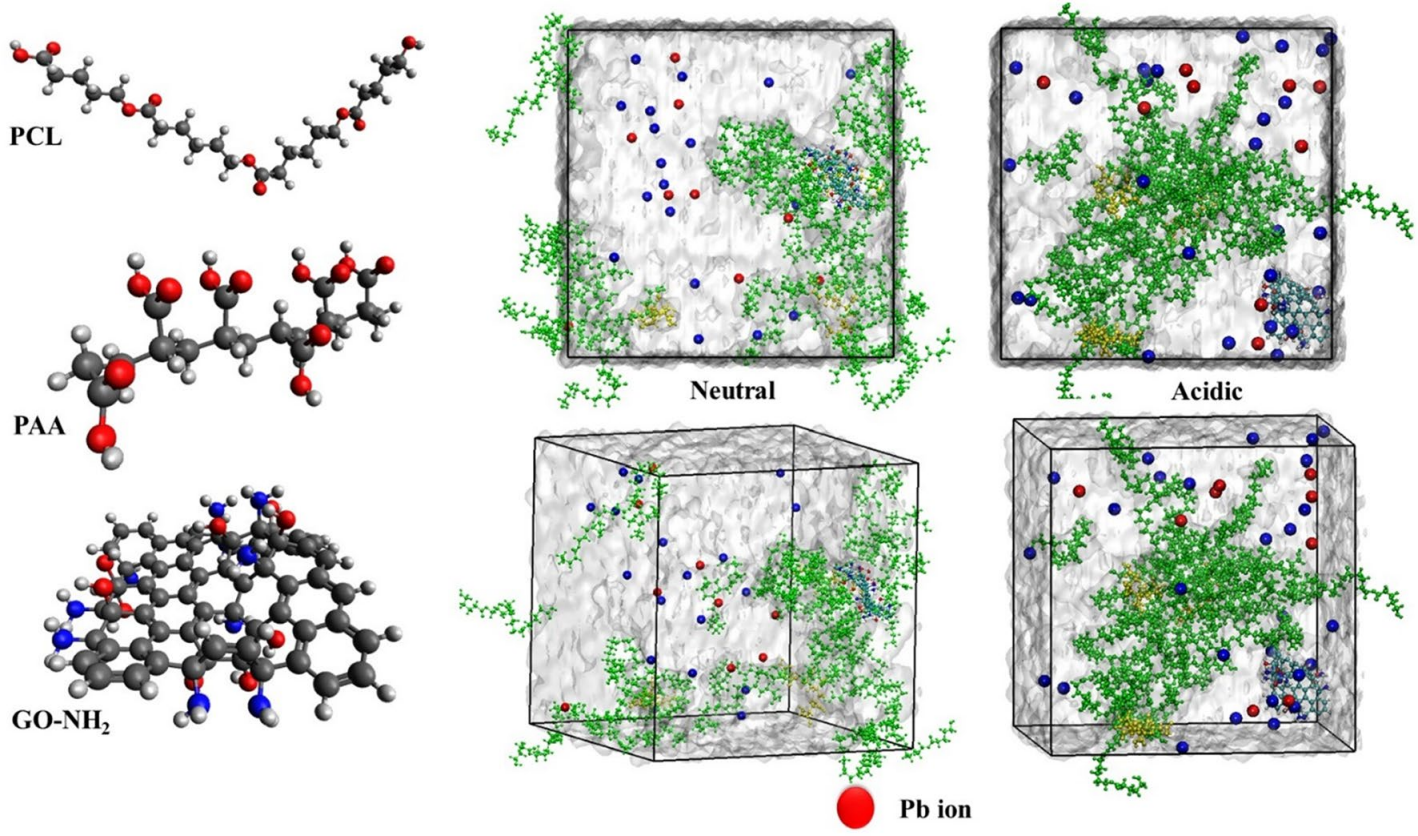
Arrangement and distribution of polymers, together with the extent of lead metal absorption under both acidic and neutral conditions.

However, when GO contains NH_2_ groups, it undergoes a transformation into NH^3+^ in the presence of acidic circumstances**. **Figure 14 displays the arrangement and distribution of polymers, together with the extent of Pb^2+^ metal adsorption under both acidic and neutral environments. Observing the simulation box reveals that the dispersion of polymers is more pronounced in neutral circumstances. Furthermore, the adsorption and positioning of Pb^2+^ metal exhibit a stronger inclination under these conditions. However, in an acidic environment, as seen in the simulation box, polymers exhibit a higher density. Additionally, the Pb^2+^ ion dispersion is considerably greater. Conversely, the NH_2_ functional groups present on the surface of graphene oxide undergo a conversion into NH_3_^+^ groups. This conversion results in the Pb^2+^ ion being positioned at the furthest distance from the polymers and ultimately determines the final composition.

### Adsorption of lead metal by NFs in apple juice media

To address the health hazards associated with heavy metals and their occurrence in food, it is essential to devise techniques for eliminating these metals from food systems. Significant quantities of electrospun NFs have been used to extract and adsorb substantial amounts of heavy metals from water and wastewater. The use of adsorption systems in the field of food is innovative and groundbreaking. The investigation focused on the adsorption of Pb^2+^ metal in apple juice. NFs with ideal conditions for Pb^2+^ metal adsorption in aqueous systems have been used to adsorb lead metal in apple juice medium.

To achieve this objective, 100 µl of a Pb^2+^ solution with a concentration of 100 parts per million (ppm) have been introduced into the apple juice. A fraction of the solution is subjected to acid digestion without the inclusion of fibers and then introduced into the device to quantify the metal content before adsorption. A nanofiber adsorbent was introduced into a quantity of apple juice solution. After the allotted time for adsorption had elapsed, the solution underwent acid digestion, and the leftover Pb^2+^ metal was then put into the ICP apparatus. The examination of heavy metal adsorption from apple juice revealed that the addition of Pb^2+^ metal to the juice resulted in around 70% adsorption of the Pb^2+^ metal. The presence of other chemicals in juice composition decreases the likelihood of Pb^2+^ metal adsorption on the surface and fiber sites.

The findings indicate that the combination of acid digestion and the addition of Pb^2+^ to apple juice, followed by adsorption, resulted in a significant 76% reduction in heavy metal content in the solution. This is because the solution, after digested, has a very low and acidic pH. Consequently, the solution exhibits a high concentration of H^+^ and H_3_O^+^ ions, resulting in a decrease in adsorption activity.

## Conclusions

Electrospun NFs have a high potential to remove heavy metals according to their characteristics. In this study, PCL polymer was used as a suitable and safe material for sequestering Pb^2+^ metal. PAA and GO were used to improve the properties and increase the adsorption efficiency of heavy metals in combination with PCL. The investigation of its morphology and assessment of its characteristics supported this choice. The final nanofibers had a size of 216 nm, suitable mechanical properties, a semi-crystalline surface, high porosity, a high surface-to-volume ratio, suitable hydrophobic properties, and were biocompatible and degradable. The resilience of PCL fibers enables their multiple applications in lead metal adsorption. To enhance the efficiency of Pb^2+^ metal adsorption, the NFs were composed of both PAA and GO with amine functionalities. The FTIR analysis confirmed the presence of these substances in the final composition of the NFs. The examination of Pb^2+^ metal adsorption showed that, regardless of the tested settings and parameters, the adsorption rate exceeded 80%. The adsorption isotherm model of PCL/PAA/GO NFs in the adsorption of Pb^2+^ metal follows the Langmuir model, and the reaction kinetics follow the pseudo-second-order. PCL/PA/GO NFs have shown adsorption of over 80% in four consecutive cycles. The use of a minimal amount of adsorbent has proven to be highly effective in adsorbing substances. In this case, specially designed NFs with a high-water adsorption capacity were utilized to extract Pb^2+^ metal from apple juice. These NFs exhibited an impressive adsorption rate of 70%. Moreover, these NFs possess non-toxic properties and demonstrate excellent functional efficiency. As a result, they can be used as adsorbers and sensors for heavy metals, as well as for detecting water and food contaminants.

## Data Availability

The data that support the findings of this study are available from the corresponding author upon reasonable request.
